# Understanding how the university curriculum impacts student wellbeing: a qualitative study

**DOI:** 10.1007/s10734-022-00969-8

**Published:** 2022-12-02

**Authors:** Rebecca Upsher, Zephyr Percy, Lorenzo Cappiello, Nicola Byrom, Gareth Hughes, Jennifer Oates, Anna Nobili, Katie Rakow, Chinwe Anaukwu, Juliet Foster

**Affiliations:** 1 King’s College London Institute of Psychiatry Psychology and Neuroscience, London, UK; 2 University of Derby, Student Wellbeing, Derby, Derby UK; 3 University of Surrey, School of Health Sciences, Surrey, UK

**Keywords:** University, Wellbeing, Mental health, Qualitative, Curriculum

## Abstract

There is increasing pressure within universities to address student mental health. From a whole university or settings-based perspective, this could include curriculum-embedded approaches. There is little research about how this should work or what approaches might be most effective. Semi -structured interviews were conducted with fifty-seven undergraduate students from five disciplines (Psychology, English studies, Nursing, International Politics, and War Studies) to understand students’ perspectives. Students reflected on wellbeing module content and, more broadly, on curriculum processes (teaching, pedagogy, assessment) within their degree. Reflexive thematic analysis was applied to transcripts, generating three themes: embedding wellbeing in the curriculum; assessment, challenge, and academic support; and social connection and interaction. The findings provide evidence for teaching, pedagogy, and assessment practices supporting higher education student wellbeing. These align with recommended good teaching practices, such as considering appropriate assessment methods followed by effective feedback. Students saw the benefits of being academically challenged if scaffolded appropriately. Strong peer connection, teacher-student interaction, and communication were crucial to learning and wellbeing. These findings provide implications for future curriculum design that can support learning and wellbeing.

## Introduction

Internationally, university students have a high prevalence of mental distress (Auerbach et al., 2018; Tabor et al., 2021). Many students are transitioning from adolescence to adulthood when entering Higher Education (HE): this is a vulnerable period as most mental health issues have their onset before age 24 (Reavley & Jorm, 2010). Demands come from becoming more financially and socially independent, exploring new relationships, parental autonomy, mastering new ways of learning, and adapting to a different social environment while establishing career plans (Macaskill, 2013). The pressure to meet these demands is stressful and can negatively impact mental wellbeing and interfere with the capacity to learn (Hamaideh, 2011). Poor mental wellbeing affects attendance, engagement with assessments, concentration, self-efficacy, motivation, and self-confidence (Quinn et al., 2009). Mental health services can help alleviate this rising prevalence (Broglia et al., 2018). However, in referring students for counselling, academics may be inappropriately medicalising their distress, implying the cause of and solution to distress lies with the individual student (Ecclestone, 2020). With a high prevalence of mental distress in our universities, it is appropriate to ask whether the university environment contributes to this distress. The environment is the perspective taken by setting-based models, such as the University Mental Health Charter (Ashton, 1998; Hughes & Spanner, 2019). The University Mental Health Charter advocates for a ‘whole-university approach’ (Hughes & Spanner, 2019) where health promotion addresses wellbeing through organisational, structural and environmental changes (Dooris & Doherty, 2010; Hughes & Spanner, 2019). This whole-university approach is preventative and universal (Thorley, 2017).

“Learning”, including teaching and assessment, is one of the four themes outlined in the University Mental Health Charter (Hughes & Spanner, 2019). The classroom, be it online or face to face, is a guaranteed contact point between teaching staff and university students (Houghton & Anderson, 2017). Therefore, pedagogic approaches adopted by academics impact all students. When asked what universities can do to improve wellbeing, many student recommendations relate to the curriculum (e.g. changes to course design, teaching practices or assessment) (Baik et al., 2019). Students identify how they are taught and assessed as a potential source of distress (Sampson et al., 2022). In this context, academics are embedding wellbeing activities into their curriculum and considering pedagogy from the perspective of wellbeing.

The Higher Education Academy (HEA) has suggested several strategies to embed wellbeing in the curriculum; these include relating course content to wellbeing and promoting teaching strategies fostering peer connection, active learning, and autonomy (Houghton & Anderson, 2017). Other recommendations include redesigning the curriculum to reduce undue stress, enhancing the provision of guidance and support, designing inclusive assessment methods (Burgess et al., 2009), and developing study skills (Putwain et al., 2013). However, evidence supporting interventions in adapting curriculum, pedagogy, and assessment design is limited (Worsley et al., 2020). Two systematic reviews examining setting-based, curriculum-embedded approaches supporting university student wellbeing found inconclusive results due to low internal validity, lack of controls, and poor and inconsistent reporting (Fernandez et al., 2016; Upsher et al., 2022b). The sector, therefore, requires further evidence to develop interventions and evaluate effectiveness.

Before further development and testing of interventions, the sector needs a more robust understanding of the existing curriculum-embedded approaches and students’ perspectives of these. To address this gap, we interviewed undergraduate students to explore their perceptions of how curriculum, pedagogy, and assessment design impact wellbeing.

## Methods

This study informs a larger project called “Education for Mental Health” (Hughes et al., 2022), a national online toolkit for academics to support student learning and wellbeing through curriculum, pedagogy and assessment.

### Design

This qualitative study had a critical realist ontology with objective epistemology, the research paradigm was post-positivist (Clark & Braun, 2013). The positionality was empathetic reflexive judgement, and researchers had a subjective spectator (lead researcher; RU) and co-creator (student researchers) position within the data. Student researchers acted as co-creators in the research process, informing data collection, analysis, and dissemination, which was essential in understanding the student perspective (Piper & Emmanuel, 2019). These students had qualitative training and mentoring from the research team (RU, NB, JF). Academics from a large London university were encouraged to collaborate with the project if they were adapting their teaching or curriculum to enhance student wellbeing. Online semi-structured one-to-one interviews and focus groups of undergraduate students were conducted. Students were recruited between September 2020–April 2021 and were interviewed once^1^ (see footnote 1). University ethical approval was obtained.

### Participants

Undergraduate students were recruited from modules that incorporated a student wellbeing element. Modules were not developed explicitly for this study. Modules could include any undergraduate student in any year or subject, delivered via any mode (face-to-face or online), compulsory or optional, credited, or non-credited. Relevant module leaders interested in collaborating responded to the research team via university staff forums. Module leaders from five undergraduate programmes collaborated: Psychology, English studies, Nursing, International Politics, and War Studies. A convenience sample of fifty-seven students across these modules (see Table 1 for participants’ demographics) were recruited via module forum posts, signposting from module leaders, short seminar presentations from the research team, university research participation systems, and student representatives. Table 2 summarises modules according to the TIDieR guidelines (Hoffmann et al., 2014).

**Table 1:** Participant demographics

	*N* (%) Departments					
Variables	Total	Psychology	English Studies	Nursing	International Politics	War Studies
Age						
17–20	37 (64.9)	13 (76.5)	7 (63.6)	3 (60)	6 (42.8)	8 (80)
21–24	17 (29.8)	4 (23.5)	3 (27.3)	-	8 (57.2)	2 (20)
25 +	3 (5.3)	-	1 (9.1)	2 (40)	-	-
Gender						
Female	42 (73.7)	16 (94.1)	10 (90.9)	2 (40)	9 (64.5)	5 (50)
Male	14 (24.6)	-	1 (9.1)	3 (60)	5 (35.7)	5 (50)
Other	1 (1.7)	1 (5.9)	-	-	-	-
Ethnicity						
Any other white background	18 (31.6)	7 (41.3)	-	-	6 (60)	5 (35.7)
Scottish/English/Welsh/Northern Irish/British	8 (14)	1 (5.9)	2 (18.2)	2 (40)	2 (20)	1 (7.14)
Indian	6 (10.5)	4 (23.5)	1 (9.1)	-	-	1 (7.14)
Pakistani	6 (10.5)	1 (5.9)	2 (18.2)	-	1 (10)	2 (14.3)
African	3 (5.3)	1 (5.9)	-	2 (40)	-	-
White and Asian	3 (5.3)	-	-	1 (20)	-	2 (14.3)
Bangladeshi	2 (3.6)	1 (5.9)	1 (9.1)	-	-	-
Prefer not to say	2 (3.6)	-	1 (9.1)	-	-	1 (7.14)
Any other mixed background	1 (1.7)	-	-	-	-	1 (7.14)
Caribbean	1 (1.7)	-	1 (9.1)	-	-	-
Chinese	1 (1.7)	-	-	-	-	1 (7.14)
White and Black African	1 (1.7)	-	-	-	1 (10)	-
Other ethnic group	5 (8.8)	2 (11.8)	3 (27.2)	-	-	-
Status						
EU	15 (26.3)	6 (35.3)	1 (9.1)	-	5 (35.7)	3 (30)
Home (UK)	33 (57.9)	10 (58.8)	8 (72.7)	5 (100)	5 (35.7)	5 (50)
International	9 (15.8)	1 (5.9)	2 (18.2)	-	4 (28.6)	2 (20)
Mode						
Face-to-face	10 (17.5)		2 (18.2)	-	8 (57.2)	-
Online	30 (52.6)		9 (81.8)	5 (100)	6 (42.8)	10 (100)
Multi-modal	17 (29.9)	17 (100)				
Year						
1	22 (38.6)	3 (17.6)	4 (36.4)	5 (100)	-	10 (100)
2	18 (31.6)	8 (47.1)	4 (36.4)	-	6 (42.8)	-
3	17 (29.8)	6 (35.3)	3 (27.2)	-	8 (57.2)	-

**Table 2:** Summary of modules that incorporated a student wellbeing element according to TIDieR checklist

Discipline; module name	Number of students taking module; year of study	Why: wellbeing approach	**What**: module description	Who provided (fidelity)	How/Where	When and how much
Psychology: Graduate Attributes	*N* = 296; All years	Academic-based strategy	Sessions on core study skills, digital literacy, skills for mental wellbeing, communication skills, and building a career. Optional. Non-credited	Senior lecturers and teaching fellows in Psychology department	On-campus and online; synchronous and asynchronous (LinkedIn learning videos)	Two semesters; 20 sessions weekly
English: Skills & Support for your Degree	*N* = 220; All years	Academic-based strategy	Skills development for degree, career support to improve wellbeing. Optional. Non-credited	Senior lecturers in English department	On-campus and online; synchronous	Two semesters; 22 sessions weekly
Nursing: Wellbeing in London	*N* = 36; ^1st^ years	Five ways to wellbeing	Supports students to engage in the 5 ways to wellbeing: Keep moving, invest in relationships, never stop learning, give to others, savour the moment. Optional. Credited	Lecturer in nursing education	Online; synchronous	One semester; 6 sessions weekly
International Politics: Issues in International Politics	*N* = 89; ^2nd^ years	Curriculum infusion	Teaches theoretical and empirical content regarding international relations. Sessions are structured around human emotions. Optional. Credited	Teaching fellow and graduate teaching assistant	On-campus and online; synchronous	One semester; 10 sessions weekly
War Studies: Study Skills Extended Induction	*N* = 300, ^1st^ years	Academic-based strategy	Supports the transition to university. It is organised around three themes: knowledge building, community building, and skills building. This module has four components to support students: whole cohort lectures, small group seminars, peer mentoring and drop-in sessions. Compulsory. Non-credited	Lecturers/Graduate teaching assistant	Online; synchronous	Two semesters; 20 sessions weekly

### Topic guide

The research team developed the topic guide. It was assessed for face validity through pilot interviews with undergraduate nursing students (*n* = 4) who were not included in the study sample and was revised accordingly. The questions focused on aspects of the module related to wellbeing, for example, “In the context of mental wellbeing, what aspects of the module did you find challenged you most?” However, often students shared experiences in the context of their overall degree experience, and it was not entirely possible to disentangle students' experience of one module over the curriculum of their entire degree.

### Data collection

Recruitment adverts signposted students to an online survey (Qualtrics) containing an information sheet and consent form. The survey asked students for their email addresses and to indicate their availability for an interview. The survey informed students that the UK national body, Office for Students, funded the study and that the purpose was to explore perceptions of how the curriculum impacts wellbeing and experiences of engaging in a module that incorporated a student wellbeing element. Participants were unknown to researchers prior to participation.

A research associate (RU; PhD), an MSc Mental Health Studies student (ZP), and six BSc Psychology students (LC, CA, EB, HA, KA, MC) conducted interviews and focus groups. Interviewers listened to each other’s first interviews, and ongoing discussions of interview techniques allowed for fidelity assessment and minimised interviewer bias.

Interviews and focus groups were video recorded on Microsoft Teams, then transcribed verbatim and anonymised. Focus groups contained two to four students. Participants were emailed a £10 shopping voucher incentive. Interviews lasted around 30 min, and focus groups around 1 h. Students were advised to email the research team (RU) if they had further thoughts after the interview/focus group.

### Data analysis

NVivo software managed anonymised transcripts (NVivo, 2020). Eight researchers who conducted the interviews applied reflexive thematic analysis to the dataset (Braun & Clarke, 2006; Braun & Clarke, 2019):

1. Data familiarisation: Researchers familiarised themselves with the data by writing notes of initial impressions after interviews/focus groups, listening to audio files, and reading and making notes from transcripts.
2. Generating initial codes: Following reviewing notes from stage one, all transcripts were coded by the researcher who conducted the interview/focus group and a second researcher (RU, ZP, LC) on the team (who had interviewed students from a different subject cohort). This process was not “consensus coding” but rather encouraged a reflective and reflexive approach to coding, that acknowledged the diverse research team. Coding was inductive, hence data driven. Where appropriate, some data extracts were coded more than once if interpreted as having more than one meaning. Each researcher had their NVivo file and a list of codes from coding their respective transcripts. Team meetings discussed what code lists comprised of and what data evidenced those codes. This phase ended with a list of codes identified across the dataset.
3. Generating themes: This phase (and phase 4) was conducted by three researchers (RU, ZP, LC). Codes were then sorted into potential themes; this was facilitated by NVivo, i.e., creating potential labels for themes and moving the codes (with associated data extracts) into these headings. From here, subthemes were generated under each theme.
4. Reviewing themes: This phase involved reviewing the data under each theme and evaluating whether it adequately supported the theme. In instances where this was not the case, subthemes could be collapsed or merged, and data extracts were re-coded and moved into different or new subthemes. This resulted in a thematic map. Then, we evaluated whether themes accurately represented the entire dataset.
5. Defining and naming themes: This phase involved all members of the authorship team. This phase involved defining the themes and agreeing theme and subtheme names. This phase allowed for further refinement of themes/subthemes.
6. Producing the manuscript: Once themes had been defined, the analysis could be written-up with analytic narrative alongside data extracts that support points being made.

## Results

Three themes were generated: (1) embedding wellbeing content in the curriculum; (2) assessment, challenge, and academic support; and (3) social connection and interaction. The first theme encompasses student perceptions of including wellbeing content within the curriculum, but students often referred to teaching practices that influenced their wellbeing. Therefore, the remaining themes focus on these teaching practices, referring to the module participants were recruited from and their overall degree experience.

Following each quote, in brackets, we refer to which subject cohort the student belonged to and which mode of study. Modes included online, face-to-face, or multi-modal (online and face-to-face).

### Embedding wellbeing content in the curriculum

This theme explored students’ perspectives on embedding wellbeing content in the curriculum and the impact of doing so.

#### Perspectives on embedding wellbeing in the curriculum

Students from every cohort had positive perceptions about the wellbeing content in their module:

“*Our lecturer does a really good job of picking the week’s topic. Each week is named after an emotion, so this week is Anxiety in International Politics, which is super interesting and cool*.” (International Politics, online).

Students across cohorts found that certain content indirectly positively impacted their wellbeing, for example, learning about university societies (Psychology, English Studies, War Studies, Nursing), placement and study abroad opportunities (Psychology, War Studies), applied news (War Studies, International Politics), physical activity (Nursing), and academic skills development (all cohorts). Academic skills often included content that was useful for their future career:

“*I think for the ones that prepared you to do something that might seem quite daunting, like applying for internships or writing your CV and knowing what you are doing, I think those impacted me in the way that after them I knew a little bit more about what I was doing*”. (Psychology, multi-modal).

A minority of participants felt that student wellbeing should either not be addressed within the curriculum or should be optional. Some of these students explained that not everybody needs wellbeing support and that resources should be for those who need it:

“*I think it’s not about having it in your curriculum. It’s more about giving the resources for someone who might need them. Not necessarily everyone has to use the resources but they have to be there all the time*”. (International Politics, online).

Some students suggested that embedding wellbeing support within the curriculum would not be necessary if students were not struggling:

“*I’m good mentally. My wellbeing, I feel like it’s good, I feel like it’s not really for me as much as it is for other people*”. (Psychology, multi-modal).

This could be explained by some Psychology and War Studies students explaining taking personal responsibility (i.e. outside of university) for their wellbeing and that it should not be supported within the university curriculum:

“*I can ask for counselling or group therapy so I don’t think it’s the module leaders’ concern really*”. (War Studies, online).

#### Impact of including wellbeing content in the curriculum

Students were asked their perspectives on embedding wellbeing support within the curriculum. The most common positive responses across cohorts alluded to positive wellbeing being essential for learning:

“If you have got really bad mental wellbeing at the time, you are not really going to be able to learn and function as best as possible at uni. It’s quite an important place to start off with”. (nursing, online)*.*

Secondly, students often replied that wellbeing support within the curriculum is vital towards buffering academic stress*:*

“*I think it’s really important that there’s a wellbeing component because it can make people feel less stressed and it’s really important*”. (Psychology, online).

However, some Psychology, War Studies and English Studies students struggled to see the connection between module content and impact on wellbeing:

“*I don’t really see how this module affects my mental wellbeing... For example, I remember we had a lecture about how we should take notes... But I wouldn't relate it to my mental wellbeing, and rather I would just say it’s enabled me to work better”. (War Studies, online)*.

Therefore, it might be important for teaching staff to clearly and explicitly communicate the intended outcomes:

“*I don’t think I was really aware of the outcomes. I realised after I was done with it*…” (Psychology, multi-modal).

Some Psychology, English Studies and Nursing students felt that departmental wellbeing support is more beneficial than university level support that is not discipline specific. However, even within department-level support, students described problems. For example, International Politics students described individual module support as not being enough as this level of support was not offered by all teaching staff within their department:

“*The only piece of feedback I would give is to keep doing what [the module leader] is already doing and maybe try to promote it with others…Seriously, if you met some of our other module conveners you would understand why…I feel like it is really valuable for mental wellbeing*”. (International Politics, *face-to-face*).

### Assessment, challenge, and academic support

This theme explores students’ views of appropriate assessment methods and support required to undertake assessment whilst limiting negative impact on wellbeing.

#### Assessment methods

Assessment methods differed across modules. Nursing and International Politics were credited modules and assessed via 100% essay-based coursework and 50:50 essay-exam, respectively. English Studies and War Studies modules were non-assessed and optional, valued by some students as they did not add pressure to their busy courses:

“*It was nice to have something you can just choose to watch or choose not to, so it does not add to the stress of an already quite busy course*”. (War Studies, online).

The Psychology module was optional too, but it was assessed via attendance and completion of online quizzes, which was considered more enjoyable by many students compared to traditional assessments:

“*So, just completing the quizzes at the end it was a nice change of pace, I would say, than the classic assignments and exam. So yes, I liked it for this type of module*”. (Psychology, online).

Psychology, war studies and international politics students found that stress in their degree comes from having multiple deadlines overlapping:

“*Definitely when there are a lot of assignments in the same week and the same period, that’s really stressful*”. (War Studies, online)*.*

Further, assessments sometimes elicited fear responses due to the threat and potential consequence of failing:

“*I was so stressed and it hurt so much to get a high grade in semester one that I wasn’t going to do that again. I was like I don’t want to be in that place again where I screwed it up, so it meant you do what's going to be good for your mental wellbeing instead of what you want to do for the actual course*”. (International Politics, *face-to-face*)*.*

Students’ suggestions regarding embedding wellbeing into assessment design included; clearer guidance for assessment (Psychology, International Politics), guaranteed access to support those in need (Psychology, International Politics, English Studies, Nursing), and adequate time between assessments (International Politics):

“*I would say that a lot of the helpfulness around wellbeing within the modules has come from clarification and flexibility with assignments…that kind of patience and reiteration of what needs to be done and when is definitely helpful for wellbeing*”. (War Studies, online).

#### Level of challenge

International Politics and Nursing students were optimistic about appropriately challenging but worthwhile content:

“*We’re students and we’re getting prepared for our future jobs and careers, so I think it’s good to have these challenges, but having your professor and the adequate resources to help you has a positive impact on the students*”. (International Politics, *face-to-face*).

Whilst content that was ‘too difficult’ or ‘too heavy’ was found to be unhelpful:

“*There is a weird amount of reading that we have to do. I understand it is university, it is not school anymore…Core reading can be a lot. I find that not good for my wellbeing*”. (Psychology, multi-modal). 

This is supported by students across cohorts describing academic pressure as the most likely contributor to disengagement:

“*I would definitely say the stress and the pressure that, unfortunately, very often occur in your studying because you have deadlines, obviously; you feel the pressure of being evaluated on what you are studying and that takes away the curiosity part*”. (International Politics, online).

#### Academic guidance and feedback

Students across cohorts valued easy-to-access resources and firm guidance and support to prepare them for assessment:

“*He [Module leader] was very straightforward in preparing us for the essay. He made his expectations clear so credit where credit is due on that one*”. (International Politics, *face-to-face*).

Conversely, a lack of clear guidance led to feelings of insecurity for students across all cohorts:

“*There’s not much validation on whether or not I’m doing the right thing because it’s so abstract that it obviously gives you a lot of freedom, but at the same time it makes me feel very insecure because I don't know if I’m doing anything correctly at all*”. (English Studies, online). 

Where International Politics students did not find assessment feedback helpful, for some it had a negative impact on wellbeing:

“*Another thing I suppose is when you don’t get as much feedback on what you’ve done as well, so there have been some instances where the feedback has been a bit unclear and that’s also I think unconducive to wellbeing because you’re not sure what the next steps are that you have to take*”. (International Politics, online).

As a consequence, War Studies, International Politics and Nursing students called for stronger assessment scaffolding:

“*Maybe more example pieces of work, just to understand what kind of standard is required of us*”. (War Studies, online).

## Social connection and interaction

Students valued social connection with peers and teaching staff as supportive of their wellbeing. In addition, teaching staff's attributes in their communication with students was an essential enabler to wellbeing.

### Teacher-student interaction

Students from all cohorts expressed ways that helped them connect with teaching staff, e.g., teaching staff checking in with students pre-session and post-session, weekly contact via personalised emails, and teacher interaction with smaller classroom groups:

“*In seminars we were in groups and he [module leader] would really take his time to come in with each other, so I think that helps you when you’ve got contact and a conversation with your professor*”. (International Politics, *face-to-face*).

Psychology and English Studies students appreciated activities where they could get to know teaching staff, for example, finding out more about their personal lives, such as favourite books, music or tv-shows:

“*In one of my modules the seminar leader will start off each session letting us say one thing that we really enjoyed this week, so if it’s a movie, a book, or a play or something like that... I think it’s things like that that are just a bit detached from our studies that can make us connect in a social environment because otherwise we wouldn’*t”. (English Studies, online).

War Studies students outlined clear communication as vital to positive wellbeing, e.g., clearly directives, where to find resources and messages, and when deadlines are. Psychology and International Politics students found communication with teaching staff via office hours and discussion forums were valuable tools:

“*I really liked the discussion forums that you could just ask a question and then you could hear back from the module leaders or anyone else could jump in and answer”* (Psychology, multi-modal).

Live sessions provided better connection and interaction than asynchronous sessions for some Psychology, English Studies and International Politics students:

“*I think I prefer the live sessions, though, just because I like the human touch*”. (Psychology, Online).

However, more frequently, challenges experienced by asynchronous approaches were shared, for instance, difficulties in managing or monitoring participation, misinterpretation of ideas, and delayed response:

“*If we didn’t understand something that they were saying, we couldn’t really raise our hand or anything, we had to watch the whole lecture and then email them afterwards. But even when you email them and you ask your question, sometimes it won’t really be clear as opposed when you’re in person asking*”. (English Studies, *face-to-face*).

Students from Psychology, War Studies and International Politics found copious amounts of online asynchronous communication overwhelming:

“*As a student I find myself receiving 20 emails a day with all the different briefs by different module leaders and then there are different updates by the [forum] platform as well, and just having to look at all of this and all of these emails contain information the follow-up steps, I would really think for me it kind of sets me into this trend of panic attack because it makes everything feel very claustrophobic, and you kind of lose a sense of where you’re going in a way*”. (War Studies, online)*.*

### Teacher attributes

Students’ perceptions (across all cohorts) of their teaching staff impacted their wellbeing, which featured in several ways, including making time for students and being friendly, personable, supportive, and empathetic:

“*It’s also really helpful when teachers, just in general, they are a lot more open as in they are more willing to get to know their students, or they remember your name, or they remember where you’re from, or they contact you being like, ‘I read this,’ or, ‘I thought this might interest you,’ or things like that*”. (International Politics, *face-to-face*).

Teaching staff being ‘engaging’ was another essential attribute to some students across all cohorts:

“*His [session leader] enthusiasm and willingness to push us on was helpful*”. (English Studies, online).

For students in all cohorts, teacher attributes were linked to a positive classroom environment that was non-judgemental, low pressure, and acted as a “safe space” to talk amongst peers and teaching staff:

“*Everybody was very welcoming, and everything was quite accommodating, so if you choose not to do something or you don’t feel comfortable with something, everybody understands, which is a great atmosphere to be around*”. (English, online).

However, there were accounts of less favourable teacher attributes, often conducive to negative wellbeing, for example, uncaring, not understanding student struggles, and lack of interest in teaching:

“*Some of my department, you can tell the ones who are very research-focused and focused less on their students… You just think that they don’t really care as much and that does detract from the wellbeing side of things, because when you're stressed you almost feel like they’re not going to help you, or if you go to them they’re not going to be anywhere near as helpful as other lecturers are*”. (International Politics, *face-to-face*).

### Peer connection

Students in all modules expressed that peer social connection was positively linked with wellbeing; for English Studies, International Politics and Nursing students, this was rooted in sharing experiences such as academic discipline:

*“But also, the very diverse group of students that are taking the same degree as I am, because that has been very nice. And being able to, even in this situation, talk about some of the issues with the people on my course, that has been helpful.”* (International Politics, online).

Students in all modules commented on the positive nature of group work and suggested that teaching staff should create more ways of implementing it. For Psychology, War Studies, and English Studies, smaller groups were appreciated, but for other Psychology and War Studies students, the challenges of being taught in large groups were evident:

“*It’s so awkward in the large sessions when the lecturer asks a question, and no one speaks. You just know that they are not speaking because of how big the group is. I think that makes it a bit worse for wellbeing*”. (Psychology, online).

The most positive feature of online learning described by Psychology, English Studies, and International Politics students was the function to break out into virtual rooms for group work, giving students a chance to interact with their peers. Students from all subjects reported little opportunity to talk to other students during classes:

“*I feel like it has made me less sociable than I was before because you are just staring at a screen and listening to someone for ages and I feel like emotionally I am quite drawn back from what I normally would be because I am not mixing with different people*”. (English Studies, *face-to-face*).

## Discussion

This study aimed to understand student perceptions of how the undergraduate curriculum and pedagogy impact student wellbeing. Students across five subject cohorts were recruited through modules that incorporated a student wellbeing element. This allowed us to explore views of curriculum content (i.e., content designed to improve wellbeing), in addition to students reflecting on curriculum processes (e.g. pedagogy, assessment) within the module and of their degree as a whole.

Some students in our study struggled to see the connection between the module content and their wellbeing. This goes against pedagogical research, which advises that a crucial part of scaffolding students is making explicit connections between content and learning outcomes (Hughes et al., 2022; Titsworth & Mazer, 2016). One cross-sectional study suggested that meaningful content (e.g. content with personal significance) for university students was associated with positive wellbeing (Upsher et al., 2022a). Therefore, encouraging students to make these connections and outlining intended outcomes early on could increase meaningfulness and subsequent wellbeing.

A qualitative survey of 2776 students studying in Australia found that students desired increased access to academic skills development to support their wellbeing (Baik et al., 2019). We support these findings as students reported that study skills, e.g., essay writing and referencing, indirectly enhanced their wellbeing. The findings of our study described individual module support as insufficient, i.e., all teaching staff should offer support. Other research reports the same phenomenon, including a non-randomised controlled study of modules that aimed to support student wellbeing, where module-level interventions did not reflect significant improvements in undergraduate students’ wellbeing (Upsher, 2022c). It has long been reported that universal interventions, e.g., consistent feedback and assessment practices across departments (as opposed to singular modules), are key to supporting student learning (Gibbs & Simpson, 2005) and subsequent wellbeing.

Assessments are commonly associated with stress and anxiety in HE students (Hicks-Keeton et al., 2021). In the present study, multiple simultaneous deadlines were problematic. Students’ suggestions were consistent with those noted in past research: clearer guidance for assessment, improved access to support for those who need it, and adequate time between assessments (Baik et al., 2019). However, there could be other reasons for assessments being contentious. Hanesworth et al., 2019 argue there is bias in the development and evaluation of university assessments. For example, the content within assessments is determined by educators, and their experiences and socio-cultural backgrounds influence the process. Academics can “unconsciously conflate proof of learning with a learner becoming more like them” (Hanseworth et al., 2019). In addition, assessments over rely on students adapting to the university’s style of assessment, rather than the university adapting to a diverse student cohort (Hockings, 2010). A social justice approach to assessments looks to take into consideration diversity, increase inclusivity and accessibility, that can enhance learning outcomes (Hockings, 2010). This is believed to improve individual student wellbeing as it expands opportunities for students (Nguyen & Walker, 2015). Given our study was from the student perspective, these nuances in conceptualising how assessments are built could have been missed.

Students in our study were receptive to content that was “challenging but worthwhile.” Existing research has reflected that challenging work can improve motivation over easier tasks due to increased meaningfulness and self-efficacy (Bjork & Bjork, 2011). Therefore, there seems to be a point of “desirable difficulty” where challenging content is linked to positive wellbeing if within students’ level of competency (Bjork & Bjork, 2011). Good feedback is pivotal to learning, enhancing motivation, self-efficacy and positive emotions (Kirschner & Hendrick, 2020). Feedback has often been reported as varying in quality and quantity (Ferguson, 2011), as demonstrated in the present findings where some students did not find feedback always helpful.

Consistent with previous studies, our current findings highlight the importance of considering teacher-student relationships in embedding wellbeing into the curriculum, where care, compassion, and connection are important characteristics (Riva et al., 2020). As supported by other research, communication with teaching staff via office hours and discussion forums were helpful tools for some students (Lim et al., 2017). A classroom environment that is not psychologically safe is associated with embarrassment; consequently, students might underperform academically (Turner & Harder, 2018). In our study, a “psychologically safe” environment equated to a non-judgemental, low-pressure classroom. Peer connection was also crucial for students in our study. Elements of the curriculum that facilitates peer connection can foster a sense of community and belonging, which is vital for wellbeing at university (Maunder, 2018). Poor teacher-student relationships described in the present study could result from poor staff wellbeing. University staff burnout has escalated across the UK, resulting in increasingly poor mental health due to excessive workload, external audits, short-term contracts, and progression based on short-term outcomes (Morrish, 2019). Morrish (2019) suggested that improving conditions for university staff would consequently enhance the learning environment for students.

### Strengths and limitations

Systematic reviews have quantitatively synthesised settings-based, curriculum-embedded approaches to supporting university student wellbeing (Fernandez et al., 2016; Upsher et al., 2022b, c). However, these studies lacked a qualitative inquiry to understanding students’ perspectives of such approaches. The present qualitative research is essential to the development of the university curriculum as qualitative research can help understand assumptions (Craig et al., 2008), components (Clark, 2013) and active ingredients of complex interventions (Moore et al., 2015) that pure quantitative research cannot.

Collaboration with students in educational research is essential as students have unique and valuable insights into teaching and learning experiences and should be involved in shaping their education (Cook-Sather, 2006). The strength of working with student researchers is that it embeds a student voice within the project from data collection, analysis, and data interpretation; hence, students act as co-creators of the research, and the project capitalises on student experience and understanding (Piper & Emmanuel, 2019). The current study adds to present curricular guidelines by furthering our understanding of how curriculum can enhance wellbeing (Baik et al., 2019; Burgess et al., 2009; Hughes et al., 2022).

Students’ views of on-campus and remote learning were explored across different departments, including Psychology, English, Nursing, International Politics and War Studies. This is important as students in different faculties and learning modes have different perspectives on how universities can promote student wellbeing (Baik et al., 2019). However, the student population investigated in this study is potentially biased as the students recruited were those who were willing to have a conversation about student wellbeing. Further research is needed to investigate how the curriculum impacts wellbeing in a larger and more diverse sample of students from various faculties and universities.

## Conclusion

This study highlights the importance of understanding students’ needs to support learning and wellbeing within the curriculum. Despite the different contexts, themes generated were shared by students across disciplines and often reflected the need for good teaching practices to promote positive wellbeing. Students are happy to be challenged, but this must be appropriately scaffolded. Fostering meaningful relationships with students and supporting them to develop relationships with their peers is important. This study supports the need for a setting-based approach to supporting student wellbeing at university and provides a starting point for developing future curriculum-embedded, discipline-appropriate interventions in higher education.

## Data Availability

The data presented in this study are available on request from the corresponding author.

## Footnotes

1 Students recruited in semester A (September–December 2020) were asked about their experiences of modules in the previous academic year i.e., face-to-face teaching pre-Covid-19 pandemic. Students recruited in semester B (January–April 2021) were asked about their experiences of modules in their current academic year (2020/21) i.e., online teaching during the Covid-19 pandemic.

## Declarations

The Education for Mental Health project is part of the Mental Health Challenge Competition programme, funded by the Office for Students. The Office for Students is the independent regulator for higher education in England. Their aim is to ensure that every student, whatever their background, has a fulfilling experience of higher education that enriches their lives and careers.
